# Crystal structure of *Aspergillus fumigatus* AroH, an aromatic amino acid aminotransferase

**DOI:** 10.1002/prot.26234

**Published:** 2021-09-16

**Authors:** Sharon Spizzichino, Gioena Pampalone, Mirco Dindo, Agostino Bruno, Luigina Romani, Francesca Cutruzzolà, Teresa Zelante, Marco Pieroni, Barbara Cellini, Giorgio Giardina

**Affiliations:** ^1^ Department of Biochemical Sciences Sapienza University of Rome Rome; ^2^ Department of Medicine and Surgery University of Perugia Perugia Italy; ^3^ Food and Drug Department University of Parma Parma Italy; ^4^ Present address: Protein Engineering and Evolution Unit Okinawa Institute of Science and Technology Graduate University Okinawa Japan

**Keywords:** amino acid metabolism, conformational change, fungus infections, lungs immune system, substrate specificity

## Abstract

*Aspergillus fumigatus* is a saprophytic ubiquitous fungus whose spores can trigger reactions such as allergic bronchopulmonary aspergillosis or the fatal invasive pulmonary aspergillosis. To survive in the lungs, the fungus must adapt to a hypoxic and nutritionally restrictive environment, exploiting the limited availability of aromatic amino acids (AAAs) in the best possible way, as mammals do not synthesize them. A key enzyme for AAAs catabolism in *A. fumigatus* is AroH, a pyridoxal 5′‐phosphate‐dependent aromatic aminotransferase. AroH was recently shown to display a broad substrate specificity, accepting L‐kynurenine and α‐aminoadipate as amino donors besides AAAs. Given its pivotal role in the adaptability of the fungus to nutrient conditions, AroH represents a potential target for the development of innovative therapies against *A. fumigatus*‐related diseases. We have solved the crystal structure of *Af‐*AroH at 2.4 Å resolution and gained new insight into the dynamics of the enzyme's active site, which appears to be crucial for the design of inhibitors. The conformational plasticity of the active site pocket is probably linked to the wide substrate specificity of AroH.

## 1 INTRODUCTION

*Aspergillus fumigatus* is a saprophytic ubiquitous fungus whose spores are constantly inhaled by humans. Conidia (asexual spores) are normally cleared by individuals with a healthy immune system. However, in immunocompromised hosts, *A. fumigatus* can trigger reactions such as allergic bronchopulmonary aspergillosis or the fatal invasive pulmonary aspergillosis.^1^, ^2^, ^3^ In the lungs, the fungus has to adapt to a hypoxic and nutritionally limiting environment, characterized by the limited availability of aromatic amino acids (AAAs), as mammals do not synthesize tryptophan and phenylalanine.^4^

**FIGURE 1:**
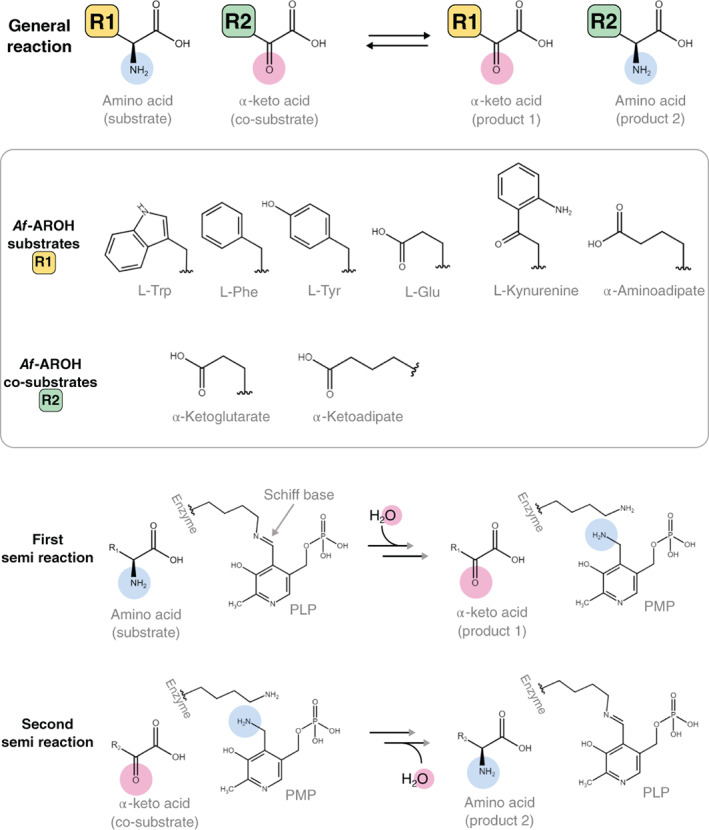
*Af*‐AroH reaction scheme—The general reaction catalyzed by *Af*‐AroH is the exchange of the amino group of an amino acid substrate with the carbonyl group of an α‐keto acid (co‐substrate), yielding the corresponding, swapped, amino acid, and α‐keto acid as products. As demonstrated in Reference 8, *Af*‐AroH can accept multiple amino acids as substrates and use both α‐ketoglutarate or α‐ketoadipate as co‐substrate (highlighted in the box). Finally, the two semi‐reactions are summarized. Each reaction implies several catalytic steps (see Reference 11 for a complete scheme). However, if the first product is not released from the active site, the enzyme cannot bind to the co‐substrate to perform the second semi‐reaction and close the catalytic cycle. All molecules are shown in the neutral form not considering the net charges

In *A. fumigatus*, L‐tryptophan catabolism is regulated by two classes of genes, *aro* and *ido*, which were both found to be highly upregulated in response to tryptophan feeding in *Aspergillus*.^5^ The *aro* genes include *aroH* (Afu2g13630) that encodes for AroH, a pyridoxal 5′‐phosphate (PLP)‐dependent aromatic aminotransferase, involved in the transamination of L‐tryptophan,^3^, ^5^ that has ∼50% sequence identity with Aro8 of *Saccharomyces cerevisiae* and *Candida albicans*.^6^, ^7^ AroH was recently biochemically characterized^8^ and displays a wide substrate specificity as observed in the *C. albicans* homologue.^9^ It behaves as an AAA aminotransferase, but also accepts L‐kynurenine and α‐aminoadipate as amino donors, meaning that this enzyme could be involved in both AAA catabolism and L‐lysine biosynthesis (Figure 1).^8^ Given its versatility, AroH likely plays a pivotal role in the adaptability of the fungus to different environmental nutrient conditions and in the release of the pro‐inflammatory aryl hydrocarbon receptor (AhR) ligands,^10^ representing a potential target in the development of innovative therapies against *A. fumigatus*‐related diseases.

We have solved the crystal structure of *Af‐*AroH at 2.4 Å resolution and gained new insight into the dynamics of the enzyme's active site, which appears to be crucial for the designing of putative inhibitors.

## 2 RESULTS AND DISCUSSION

**FIGURE 2:**
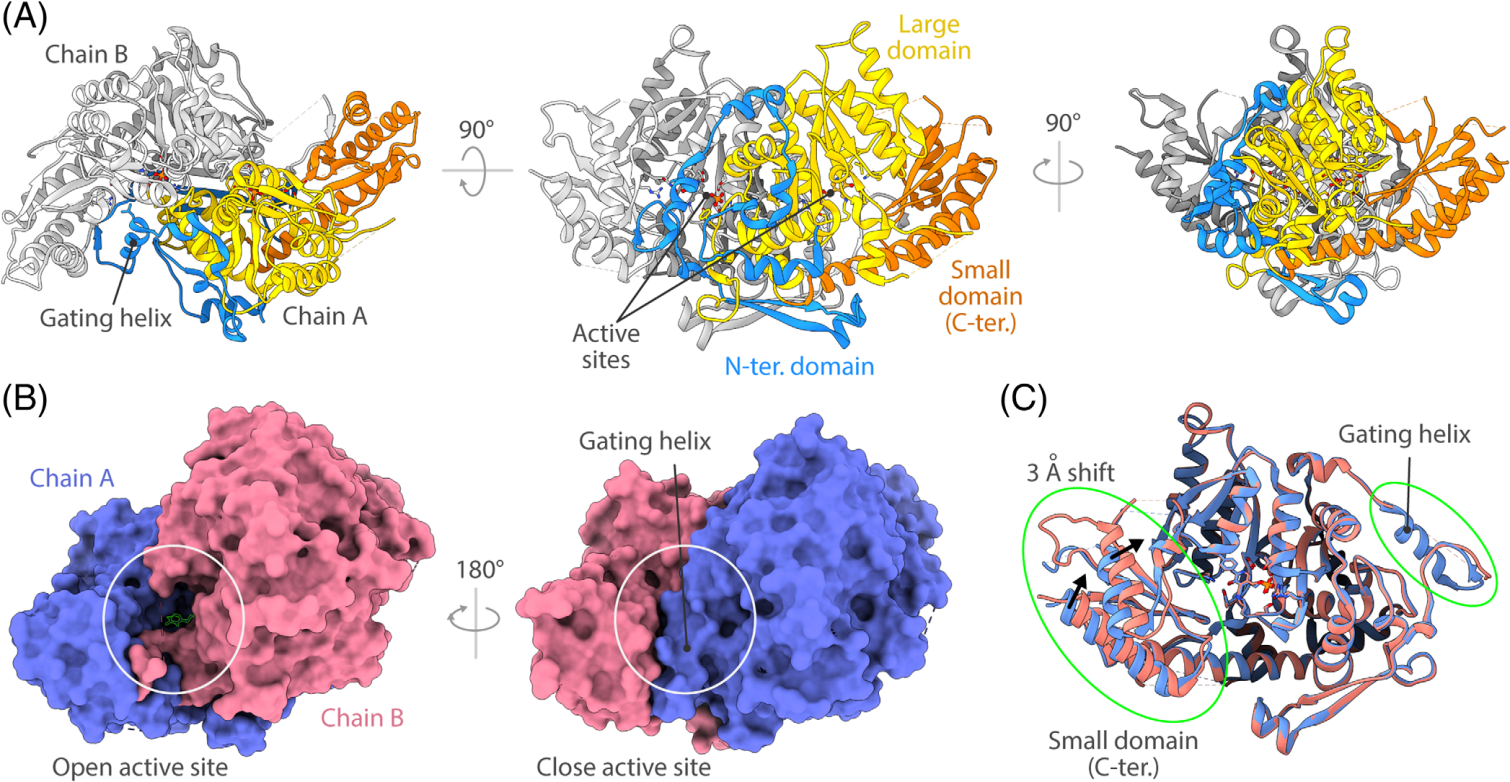
Structure of *Af*‐AroH—(A) Cartoon representation of the *Af*‐AroH dimer. Chain A is colored according to domain organization: large domain (yellow); small domain (orange); *N*‐terminus domain (blue). Chain B is colored in light gray. (B) Surface representation of the dimer. The position of the active site is highlighted by a white circle, showing the different conformations and accessibility of the active site pocket in the two subunits. The *N*‐terminus region of chain B (residues 74:85) is disordered, allowing access to active site of chain A, whereas the same region of chain A has an α‐helical fold (gating helix) and packs between the large domain and the small domain of chain B, closing the active site of chain B. (C) Superposition of the two subunits. The highlights show the gating helix region (disordered in chain B) and the 3 Å shift of the small domain going from the open conformation (chain A) to the closed conformation (chain B)

The structure of *Af*‐AroH was solved by X‐ray crystallography at 2.4 Å resolution. The proteins packed in a triclinic space group containing two biological dimers (chains: AB and CD) per unit cell and a solvent content of 46%. The data were phased by molecular replacement using the monomeric structure of the homologue from *S. cerevisiae* (PDB: 4JE5)^9^ as search model (41% identity). The two dimers superpose with a root mean square deviation (RMSD) of 0.35 Å and have no major structural or conformational differences; therefore, hereinafter, we will discuss the features of the AB dimer that displays an overall better geometry and lower average temperature factors (B‐factors). Each subunit displays the fold of type‐I PLP‐dependent enzymes, which comprises: (i) a large domain (residues 131–394), formed by seven β‐strands surrounded by nine α‐helices, that contains the active site pocket and extensively contacts the other monomer; (ii) a small domain, located at the C‐terminus of the protein (residues 395–525), that flanks the large domain; and iii) an *N*‐terminus region (residues 1–130) that contacts the partner subunit stabilizing the dimer and also contributes to the shaping of the active site pocket (Figure 2A). In both subunits, the cofactor is bound to K348 through a Schiff‐base bond. The internal aldimine has a distorted geometry due to the suboptimal distance (6.8 Å) between the Cα of the catalytic lysine and the PLP‐C4′ atom. This strain is typical of fold type‐I aminotransferases, as it causes a rotation of the PLP pyridine plane during the transaldimination step, according to what has been defined the *spring mechanism*.^11^

The two subunits of the dimer superpose with an RMSD of 0.28 Å sharing the same overall fold except at the level of the active site entrance. In particular, chain A displays a completely folded *N*‐terminus with an α‐helix that restricts the access to the active site (gating‐helix), while the same region of chain B is disordered and not visible in the electron density (residues 74–85) (Figure ). Notably, the last two α‐helices of the small domain of monomer B are shifted of about 3 Å with respect to chain A (Figure 2C). This allows for the formation of an H‐bond between R515(B) and the carbonyl group of G86(A), and a tighter packing of the small domain against the gating helix.

**FIGURE 3:**
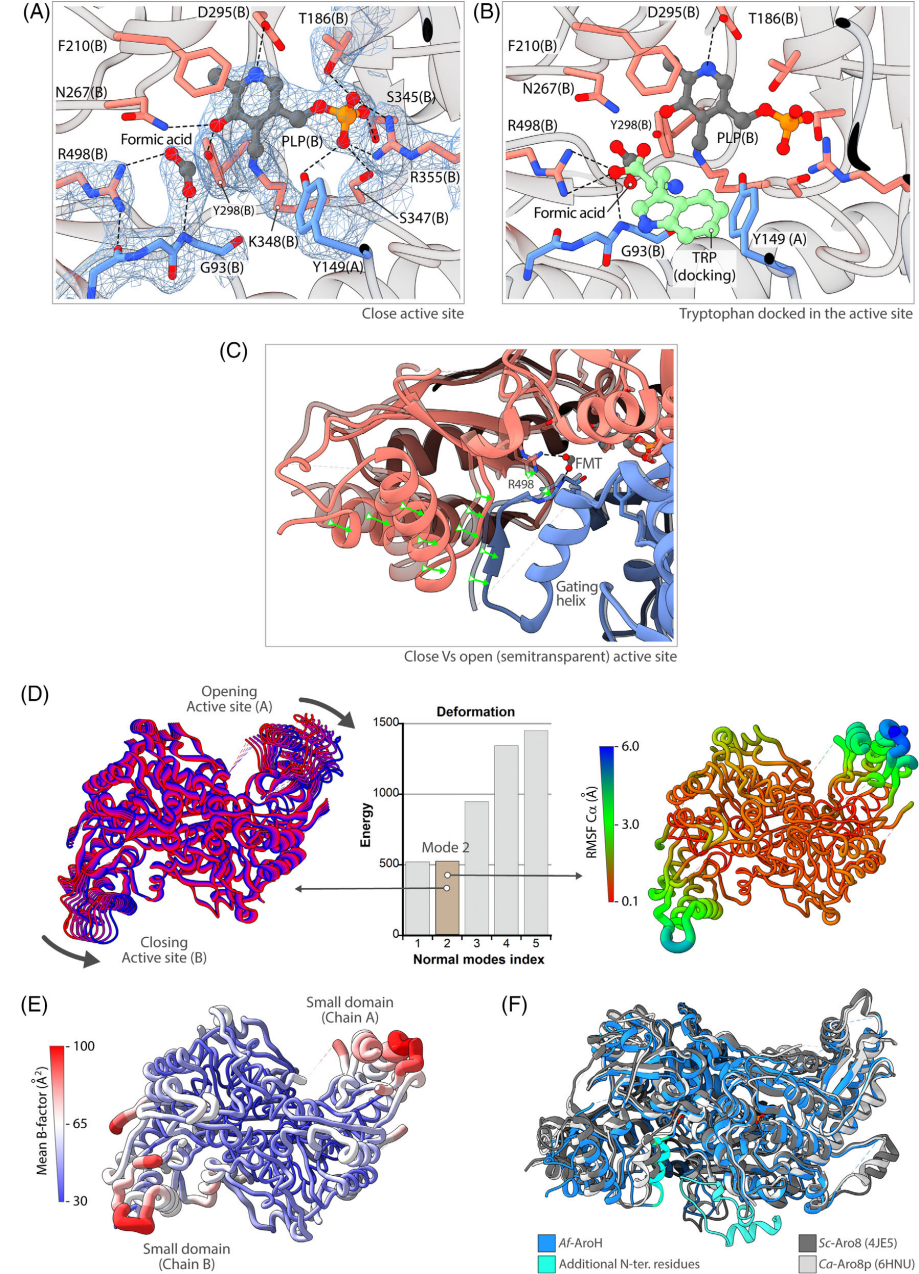
Conformational asymmetry of the active site and correlation with protein dynamics—(A) Active site of chain B binds. Key residues for PLP and ligand binding are shown as sticks: chain A (blue) and chain B (salmon). The electron density map (2*F*_o_ − *F*_c_ contoured at a 1.2 sigma value) is also shown for the internal aldimine, formate (FMT), R355, R498, Y298, and the mainchain from residues of chain A interacting with FMT. (B) Docking of a tryptophane substrate (green) in the active site. The carboxyl group docks in the same position as the formic acid molecule, interacting mainly with R498 and the mainchain from residues of chain A. (C) Superposition of closed and open active site. The green arrows highlight the shift going from the open (semi‐transparent) to the closed (no transparency) active site. (D) NMA: the deformation energy of the lowest five non‐trivial modes is shown in the middle; the first two modes represent an oscillation of the small domains in opposite and in the same direction, respectively. Mode 2 is the most interesting, and five representative models are shown on the left to illustrate the direction of the motion (from red to blue and back). The RMDS (Cα) is mapped on the structure with both radii and color that change as a function of the RMSD. The maximal displacement (up to 6 Å) is observed for the small domains. (E) Mapping of the B‐factors on the structure of *Af*‐AroH; high B‐factors correlate nicely with the dynamics described by the normal modes 1 and 2. (F) Superposition of *Af*‐AroH (blue) with the two orthologues structures: *Ca*‐Aro8p (6HNU; light gray)^14^ and *Sc*‐Aro8 (4JE5; dark gray),^9^ showing the positioning of the additional *N*‐terminus residues of *Af*‐AroH (cyan)

The conformational asymmetry of the two subunits is also reflected by the presence of a molecule of formate (FMT) only in the close active site (chain B) and not in the open one. FMT binds in the same position as the carboxylic acid of the amino acid substrates, forming two H‐bonds with R498(B) and the carbonyl group of G93(A) (Figure 3A,B). It is likely that this interaction, which slightly changes the conformation of the loop that follows the gating helix, may induce the conformational transition that closes the active site (Figure 3C). An alternative hypothesis is that the active site is transiently opened or closed, as the dimer oscillates between different conformational states. Therefore, we performed a normal mode analysis to test whether the dynamics of *Af*‐AroH is compatible with the open/close switch. Interestingly, the first two lowest non‐trivial modes correspond to a coordinated movement of the two small domains. In mode n.2, the two domains move in the same direction, resulting in the opening of one active site while closing the other one (Figure 3D and Movie S1), whereas in mode n.1, they move in opposite directions. This result is also in agreement with the B‐factor distribution of the structure of *Af*‐AroH, showing that the small domains are clearly the more dynamic regions of the protein (Figure 3E). Overall, this analysis suggests that the small domain dynamics transiently changes the active site accessibility, allowing for the access of a wide range of different substrates in the open active site, whose binding will then stabilize the closed conformation as recently demonstrated for porcine aspartate aminotransferase.^12^ It may also be speculated that this coordinated oscillation between an open and closed conformation of the active site is functional for the transaminase reaction. Indeed, the substrate binding to one subunit may induce the opening of the other active site, favoring product release in a sort of ping pong or breathing mechanism in which each semi‐reaction is alternatively carried out by one subunit (Figure 1).

Finally, it is worth mentioning that the first 50 residues at the *N*‐terminus of *Af*‐AroH, which are not present in the sequences of aromatic aminoacidic aminotransferases from other fungi, were automatically annotated as disordered.^13^ However, apart from the first 15 residues, in *Af*‐AroH, this region has a well‐defined structure, forming three small mutually perpendicular α‐helices that flank the large domain and a long loop that inserts between the gating helix and the large domain. Superposition with the other two orthologues structures available, Aro8 from *S. cerevisiae* (4JE5; 41% identity)^9^ and Aro8p form *C. albicans* (6HNU; 45% identity),^14^ reveals no major differences apart from the additional *N*‐terminus residues that are present only in the *Af*‐AroH, and for the gating helix, which appears to be shifted toward the active site pocket that in this conformation is completely inaccessible (Figure 3F).

The observed dynamics may represent a problem for the rational design of AroH inhibitors. Indeed, we performed a virtual screening campaign on AroH based on the closed conformation of the active site, which resulted in the selection of 20 compounds to be further tested *in vitro* (Table S1). Although these molecules displayed a predicted free energy of binding—Δ*G*_bind_—ranging from −50 to −35 kJ/mol (MM/GBSA approach; Table S1), none of these compounds was able to significantly inhibit AroH *in vitro* (Figure S1), indicating that the active site conformational flexibility plays an important role in substrate binding. This should be considered when planning a drug design campaign. In light of our results, in the case of *Af*‐AroH, an experimental medium/high‐throughput approach should be favored with respect to *in silico* docking methods.

## 3 MATERIALS AND METHODS

### 3.1 Protein expression and purification

AroH was expressed and purified as reported in Reference 8. Briefly, the enzyme was expressed in *Escherichia coli* (BL21‐DE3) with a C‐ter his‐tag. Expression was induced with 0.3 mM isopropyl‐β‐D‐thiogalactopyranoside (IPTG) just before exponential growth phase. Cells were grown in Luria‐Bertani (LB) media supplemented with 50 μM PLP. Temperature was lowered from 37°C to 30°C after induction, and after 15 h, cells were harvested by centrifugation, resuspended, and lysed by the addition of 0.2 μg/ml of lysozyme followed by a cycle of freeze–thaw. AroH was purified by metal ion affinity chromatography on a HisPrep FF 16/10 column (GE Healthcare). The fraction containing the protein was pooled, and the buffer was exchanged by forced dialysis to 100 mM potassium phosphate, pH 7.4, before stocking at −20°C.

### 3.2 Crystallization and structure solution

Crystallization conditions were initially screened automatically using the Crystal Phoenix robot (Art Robbins instruments) at two protein concentrations 87 and 175 μM. Small single crystals were observed in condition G4 of the Morpheus rational screen (Molecular Dimensions). Optimization of this initial hit by hanging drop vapor diffusion method produced 100 × 100 × 50 μm crystals: 1 μl of protein solution (protein concentration = 175 μM; buffer = 50 mM potassium phosphate, pH 7.4) was mixed with 1 μl of reservoir—50 mM imidazole; 50 mM 2‐(*N*‐morpholino)ethane sulfonic acid monohydrate (MES); 13.75% v/v 2‐methyl‐2,4‐pentanediol (MPD); 13.75% polyethylene glycol (PEG) 1000; 13.75% w/v PEG 3350; 2 mM Sodium formate; 20 mM ammonium acetate; 20 mM sodium citrate tribasic dihydrate; 20 mM potassium sodium tartrate tetrahydrate; 20 mM sodium oxamate—and equilibrated versus 300 μL of reservoir solution at 21°C. Crystal grew in 2–5 days. Crystals were flashed frozen in liquid nitrogen, without cryoprotection, and exposed to X‐ray at the Elettra synchrotron in Trieste (IT) on the XRD2 beamline. A total of 400 degrees were collected, with an oscillation of 0.5°, at a wavelength of 1.000 Å. Data were processed with XDS^15^ and scaled with Aimless^16^, ^17^, ^18^ at a 2.4 Å final resolution in the CCP4 suite.^19^ Space group was P1 with the following cell parameters: 65.7, 75.2, 121.6, 85.1, 88.2, and 64.8. The Matthews coefficient was 2.30 Å^3^dalton^−1^, corresponding to four molecules (two dimers) per cell and a solvent content of 46.5%. Phases were obtained by molecular replacement using the structure of Aro8 from *S. cerevisiae* (4JE5) as search model in Molrep.^20^ Iterative cycles of model building and refinement were performed with Coot^21^ and Refmac5.^22^ ProSMART^23^ was used to optimize the model geometry according to H‐bonds during the refinement cycles. Statistics for data collection and refinement are reported in Table 1. Coordinates and structure factors have been deposited in the Protein data bank with accession code 6S8W.

**TABLE 1:** Data collection and refinement statistics

Data collection	*Af*‐AroH			
Synchrotron, beamline	Elettra, XRD2			
Space group	P1			
Cell dimension; *a*–*b*–*c* (Å), *α*–*β*–*γ* (°)	64.21–75.26–121.63, 84.77–87.51–65.37			
Resolution range (Å)	47.25–2.40			
*R*_merge_	0.11 (0.78)^a^			
Half‐set correlation CC(½) (%)^b^	99.2 (59.7)			
<*I*/*σI*>	6.3 (1.7)			
Completeness (%)	97.2 (97.5)			
N. reflection (total)	298 299 (18 179)			
Multiplicity	3.8 (4.0)			
Wilson B‐factor (Å^2^)	52.2			
**Refinement**				
N. of unique reflections	74 503 (5497)			
*R*_free_ test set	3871 reflection (4.94%)			
*R*_work_/*R*_free_ (%)	24.6/29.0			
*F*_o_,*F*_c_ correlation	0.93			
Average B‐factor all atoms (Å^2^)	A	B	C	D
*Protein*	51.2	51.2	64.5	68.9
*Water*	46.5	44.5	50.2	51.5
*PLP*	45.9	48.2	71.6	59.7
*FMT*	‐	53.6	‐	47.9
Bonds RMSD				
*Length (Å)*	0.004			
*Angle (°)*	1.224			
Ramachandran plot (%, n. residues)				
*Favored*	94.5, 1799			
*Allowed*	5.2, 99			
*Outliers*	0.3, 6			

^a^ Values in parentheses refer to the highest‐resolution shell.

^b^ Percentage of correlation between intensities from random half‐data sets.

### 3.3 Molecular docking and normal modes analysis

Docking of tryptophan in the closed active site of Af‐AroH was performed with AutoDock Vina^24^ tool implemented in UCSF Chimera.^25^ The standard workflow, with default values, was used to prepare both ligand and receptor for docking. The search volume was a 20 Å cube centered on the active site. The best pose is shown in Figure 3(B). Normal modes analysis (NMA) was performed online using the WEBnma server,^26^ available at: http://apps.cbu.uib.no/webnma3/, using the dimeric structure of *Af*‐AroH (chains AB) and the Cα force field as defined in Reference 27, which assigns the mass of each residue to the corresponding Cα. The server calculates the first 200 lowest frequency modes and the relative deformation energies, eigenvalues, and the normalized squared atomic displacements.

### 3.4 Library preparation

3.4.1

Six libraries of commercially available compounds were retrieved from ChemDiv (https://www.chemdiv.com): Therapeutical Diversity Annotated Library; 3D‐Biodiversity Library; Antifungal Library; Antiparasite Library; Anti‐infective Library and Indole Derivatives. The final number of compounds was equal to 109.213. The collected compounds were prepared using LigPrep Tool of Maestro 9.1 (https://www.schrodinger.com).

### 3.5 Virtual screening workflow

The virtual screening was performed using the virtual screening workflow (VSW) implemented in Maestro9.1 (https://www.schrodinger.com). As a force field OPLS2005 was used, all the possible ionization state at pH 7.4 was generated, and 32 low energy ring conformations per ligand were generated. The docking grid box was centered on the residues lining up the protein active site. For the VSW, the default setting was used, and the 10% top ranked compounds were initially selected rescored using the MM‐GBSA methods available in VSW and visually inspected. Twenty compounds were selected to be biochemically assayed (Table S1).

### 3.6 Inhibition assays

AroH lyase activity was determined by measuring the production of L‐glutamate in a spectrophotometric assay coupled with glutamate dehydrogenase (GDH), as previously reported.^8^ The inhibitory activity of the compounds was tested upon preincubation of the enzyme at 2 μM concentration in the absence or presence of each inhibitor at 50 μM concentration for 10 min or 15 h at 25°C in 66 mM potassium phosphate buffer, pH 8.2. The residual activity of each sample was then determined by adding the enzyme‐inhibitor complex in an assay mixture containing 50 μM inhibitor, 1.4 mM L‐phenylalanine, 1 mM α‐ketoglutarate, 2 mM APAD^+^, 1.4 mM L‐phenylalanine, 1.5 mg/ml GDH, and 200 μM PLP, in 66 mM potassium phosphate buffer, pH 8.2. Each putative inhibitor was tested at least in duplicate. To promote compound solubility, inhibition assays were performed at both 0.5% and 10% (v/v) DMSO. No changes in AroH transaminase activity were observed at [DMSO] ≤10% (v/v) (Figure S1).

### 3.7 Molecular graphics

structure images, superposition, and analysis were produced or performed using UCSF Chimera,^25^ with the exception of the protein surface images (Figure 2B) that were rendered using UCSF ChimeraX.^28^, ^29^

## AUTHOR'S CONTRIBUTION

Sharon Spizzichino and Giorgio Giardina performed the crystallographic study; Mirco Dindo and Gioena Pampalone purified the protein; Agostino Bruno and Marco Pieroni performed the VSW study, and Gioena Pampalone tested the compounds *in vitro*. Giorgio Giardina, Barbara Cellini, Marco Pieroni, Francesca Cutruzzolà, Teresa Zelante, and Luigina Romani supervised the experimental work. All authors analyzed the data. Giorgio Giardina and Sharon Spizzichino wrote the manuscript with the help and critical review of all the authors.

### PEER REVIEW

The peer review history for this article is available at https://publons.com/publon/10.1002/prot.26234.

## Supporting information


**MOVIE S1** Visualization of the second lowest non‐trivial modes (mode n.2 in Figure 2) corresponding to a coordinated movement of the two small domains in the same direction resulting in the opening of one active site while closing the other one.

**TABLE S1** List of compounds obtained by virtual screening workflow selected to be assayed.
**FIGURE S1** Fold change in activity of *Af*‐AroH in the presence of the 20 best compounds from VSW.

## Data Availability

The structural data that support the findings of this study are openly available in the Protein Data Bank at https://www.rcsb.org, reference number 6S8W. Other data are available from the corresponding author upon reasonable request.
