# Supplementary material for: Crystal structure of Aspergillus fumigatus AroH, an aromatic amino acid aminotransferase
Source: Proteins. 2021 Sep 16;90(2):435–42. doi: 10.1002/prot.26234 (PMC9290597; doi:10.1002/prot.26234)
Supplement: Supplementary file 2 — TABLE S1 List of compounds obtained by virtual screening workflow selected to be assayed. FIGURE S1 Fold change in activity of Af‐AroH in the presence of the 20 best compounds from VSW. [file PROT-90-435-s002.pdf]

Supplementary information for

Crystal structure of *Aspergillus fumigatus* AroH, an aromatic amino acid aminotransferase

Corresponding author:

Giorgio Giardina, giorgio.giardina@uniroma1.it,

- **Table S1:** List of compounds obtained by virtual screening workflow selected to be assayed (below).
- **Figure S1:** Fold change in activity of *Af*-AroH in the presence of the 20 best compounds from VSW (below).
- **Movie S1:** Visualization of the second lowest non-trivial modes (mode n.2 in Fig. 3) corresponding to a coordinated movement of the two small domains in the same direction resulting in the opening of one active site while closing the other one (external file).

Supplementary figure and tables

Table S1

Compound	2-D Structure	Score (MM/GBSA ΔG binding kJ/mol)
**A01**	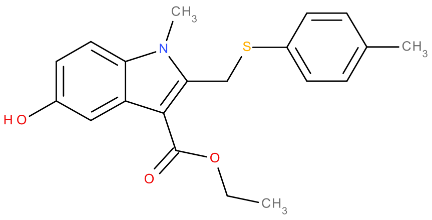	-45.8
**A02**	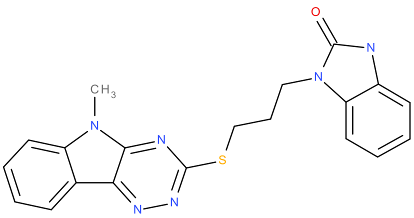	-45.2
**A03**	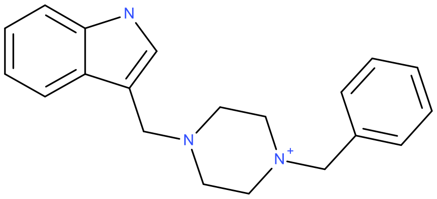	-36.1
**A04**	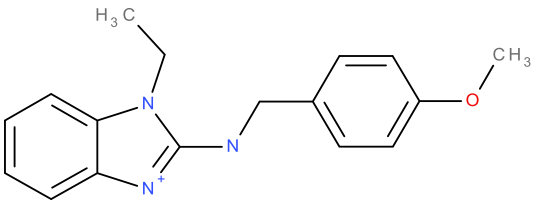	-38.5
**A05**	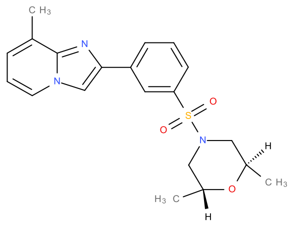	-39.3
**A06**	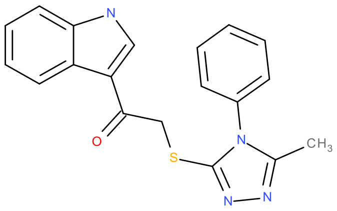	-38.6

**A07**	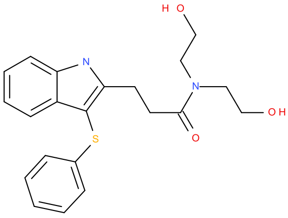	-44.9
**A08**	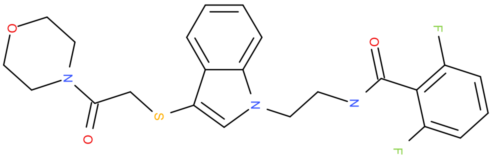	-38.5
**A09**	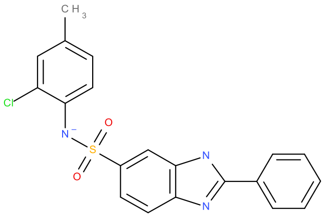	-35.3
**A10**	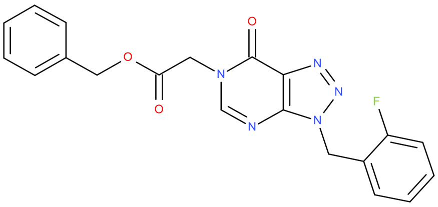	-49.2
**A11**	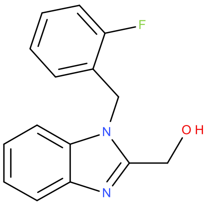	-39.7
**A12**	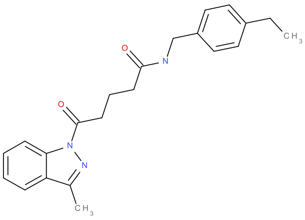	-47.5
**B01**	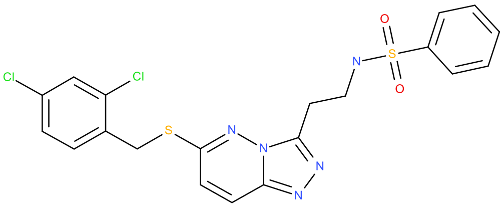	-48.5

**B02**	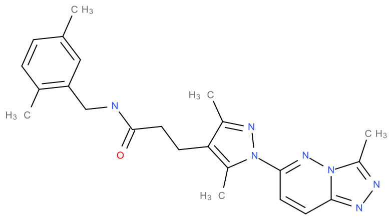	-51.7
**B03**	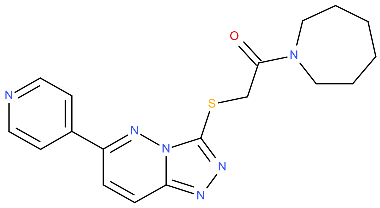	-45.7
**B04**	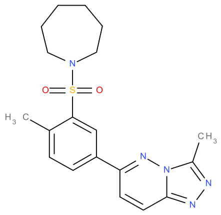	-44.3
**B05**	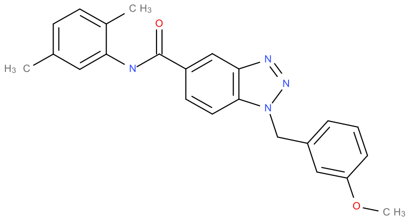	-46.6
**B06**	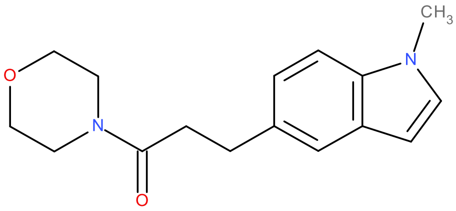	-35.8
**B07**	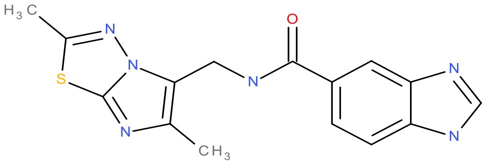	-40.6
**B08**	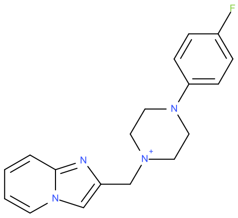	-37.8

Figure S1

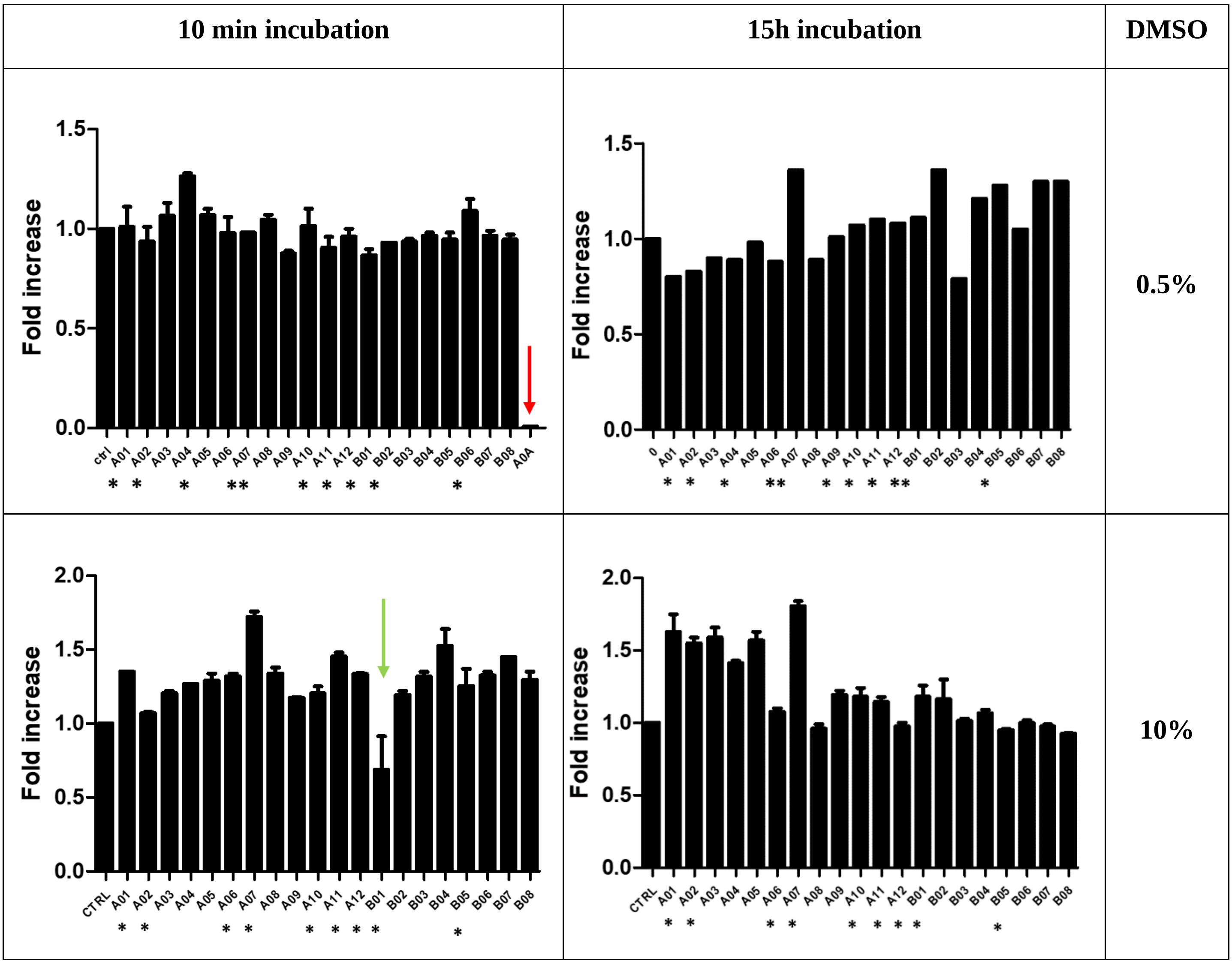

Figure S1. Fold change in activity of *Af*-AroH in the presence of the 20 best compounds from VSW – Reaction mixture contained 200 nM enzyme, 50 mM inhibitor (incubated at 10 min and 15h), 1 mM α-ketoglutarate, 2 mM APAD+, 1.4 mM L-Phenylalanine, 1.5 mg/mL GDH in 66mM KP, pH 8.2, in a final volume of 300 µL. Experiments at 10 min incubation were performed in duplicate. The assays at 10% DMSO were performed after verifying that AroH activity was unaffected by the presence of DMSO at this concentration. (*) indicates the presence of turbidity in the reaction mixture after the assay. By analyzing the different components of the mixture, we found that turbidity arises upon interaction between GDH and the inhibitor. Therefore, for each mixture a blank reaction without AroH was performed and see no signal was observed, even in compounds giving rise to turbidity. Aminooxyacetic acid (AOA) (*red arrow*) was used as positive control because it is a known PLP-dependent enzyme inhibitor. B01 inhibitor (*green arrow*) was tested in quadruplicate because the data showed high deviation, however the observed reduction in activity was not significant. The reasons underlying the observed increase in activity caused by some compounds are presently unclear, but the effect is not due to any aspecific effect on the assay procedure caused by the presence of the compounds or DMSO, as shown by the positive and negative controls.
